# Parental Attitudes Toward Standard Newborn Screening and Newborn Genomic Sequencing: Findings From the BabySeq Study

**DOI:** 10.3389/fgene.2022.867371

**Published:** 2022-04-27

**Authors:** Brittan Armstrong, Kurt D. Christensen, Casie A. Genetti, Richard B. Parad, Jill Oliver Robinson, Carrie L. Blout Zawatsky, Bethany Zettler, Alan H. Beggs, Ingrid A. Holm, Robert C. Green, Amy L. McGuire, Hadley Stevens Smith, Stacey Pereira, The BabySeq Project Team

**Affiliations:** ^1^ Center for Medical Ethics and Heath Policy, Baylor College of Medicine, Houston, TX, United States; ^2^ PRecisiOn Medicine Translational Research (PROMoTeR) Center, Department of Population Medicine, Harvard Pilgrim Health Care Institute, Boston, MA, United States; ^3^ Harvard Medical School, Boston, MA, United States; ^4^ Division of Genetics and Genomics, The Manton Center for Orphan Disease Research, Boston Children’s Hospital, Boston, MA, United States; ^5^ Department of Pediatric Newborn Medicine, Brigham and Women’s Hospital, Boston, MA, United States; ^6^ Division of Genetics, Department of Medicine, Brigham and Women’s Hospital, Boston, MA, United States; ^7^ Medical and Population Genetics, The Broad Institute, Cambridge, MA, United States; ^8^ Ariadne Labs, Boston, MA, United States; ^9^ The MGH Institute of Health Professions, Boston, MA, United States; ^10^ The Broad Institute of MIT and Harvard, Cambridge, MA, United States

**Keywords:** newborn screening (NBS), newborn sequencing, genomic sequencing, ELSI, ethics, exome sequencing, newborn genomic sequencing

## Abstract

**Introduction:** With increasing utility and decreasing cost of genomic sequencing, augmentation of standard newborn screening (NBS) programs with newborn genomic sequencing (nGS) has been proposed. Before nGS can be integrated into newborn screening, parents’ perspectives must be better understood.

**Objective:** Using data from surveys administered to parents of healthy newborns who were enrolled in the BabySeq Project, a randomized clinical trial of nGS alongside NBS, this paper reports parents’ attitudes regarding population-based NBS and nGS assessed 3 months after results disclosure.

**Methods:** Parental attitudes regarding whether all newborns should receive, and whether informed consent should be required for, NBS and nGS, as well as whether nGS should be mandated were assessed using 5-point scales from strongly disagree (=1) to strongly agree (=5). Parents’ interest in receiving types of results from nGS was assessed on a 5-point scale from not at all interested (=1) to very interested (=5). Survey responses were analyzed using Fisher’s exact tests, paired t-tests, and repeated measures ANOVA.

**Results:** At 3 months post-disclosure, 248 parents of 174 healthy newborns submitted a survey. Support for every newborn receiving standard NBS (mean 4.67) was higher than that for every newborn receiving nGS (mean 3.60; *p* < 0.001). Support for required informed consent for NBS (mean 3.44) was lower than that for nGS (mean 4.27, *p* < 0.001). Parents’ attitudes toward NBS and nGS were not significantly associated with self-reported political orientation. If hypothetically receiving nGS outside of the BabySeq Project, most parents reported being very interested in receiving information on their baby’s risk of developing a disease in childhood that can be prevented, treated, or cured (86.8%) and their risk of developing a disease during adulthood that can be prevented, treated, or cured (84.6%).

**Discussion:** Parents’ opinions are crucial to inform design and delivery of public health programs, as the success of the program hinges on parents’ trust and participation. To accommodate parents’ preferences without affecting the current high participation rates in NBS, an optional add-on consent to nGS in addition to NBS may be a feasible approach. Trial Registration ClinicalTrials.gov Identifier: NCT02422511.

## Introduction

Since starting in the 1960s as a single screening test for phenylketonuria (PKU), developed by Dr. Robert Guthrie, newborn screening (NBS) has expanded in the United States into an extremely successful mandated public health program (Koppaka 2011; CDC 2020; Baby's First Test 2021). While there are differences between states on the number and types of conditions that are screened, most states use a similar approach to mandating newborn screening, including an opt-out policy that does not require parental informed consent (Lewis and Botkin 2019). Current state-based programs can use tandem mass spectrometry to screen for over 50 different conditions to allow for presymptomatic detection, diagnosis, and treatment of conditions for which early intervention can reduce morbidity, mortality, and the social burden of disease (Cipriano et al., 2007; Therrell et al., 2015; Johnson and Wile 2017).

Building on the established success of NBS programs, some have proposed that there could be even greater public health impact if genomic sequencing (GS) were incorporated alongside current screening modalities (Genetic Alliance and District of Columbia Department of Helath 2010; Groft et al., 2017). Increases in the speed and affordability of GS have rendered it a feasible option for consideration as a population-based screening tool (Groft et al., 2017). The addition of newborn GS (nGS) to NBS programs would enable screening for more conditions than current methods alone, with the potential to benefit more families (Berg and Powell 2015; Wojcik et al., 2021). A study comparing screening results between nGS by exome sequencing and standard public health dried blood spot NBS found that the two modalities provided complementary information, with exome sequencing identifying genetic risk for conditions not detected through standard NBS in 9.4% of sequenced newborns (Wojcik et al., 2021). Additionally, although genomic sequencing has not been found to be adequately sensitive or specific to be an appropriate stand-alone screening test, combining standard NBS with nGS could increase the specificity of NBS and reduce the rates of false positives.(Bodian et al., 2016; Adhikari et al., 2020; Wojcik et al., 2021). In the same study comparing nGS and standard NBS results, nine infants were standard NBS positive but negative on exome sequencing. Seven of these infants were determined to be falsely positive on standard NBS (Wojcik et al., 2021).

Despite its potential, nGS raises both ethical concerns and implementation challenges that would need to be addressed before the integration of GS into existing NBS programs could be seriously considered (Pereira et al., 2021; Tarini 2021). In order to define and understand all relevant features of implementation, policy makers must consider input from many stakeholders, including parents. Consideration of parent perspectives is crucial in the development of ethical policies regarding the inclusion of nGS into NBS. Hypothetical parental interest in GS as a newborn screening tool has been reported (Goldenberg et al., 2014; Waisbren et al., 2015). However, opinions on many policy-relevant questions, such as whether all newborns should receive nGS, whether informed consent should be required (unlike most current NBS programs), and which types of results should be returned, have only recently started to be explored (Goldenberg et al., 2014; Lewis et al., 2018; Moultrie et al., 2020). In this paper, we present findings from surveys conducted with parents of healthy newborns who were enrolled in the BabySeq Project, a randomized clinical trial of nGS. We examine parental opinions regarding NBS and nGS universal application, parental informed consent, and types of nGS results to be disclosed that can inform discourse and policymaking regarding the addition of nGS to NBS.

## Materials and Methods

### Study Participants and Design

The BabySeq Project is a series of randomized clinical trials designed to assess the medical, behavioral, and economic impact of nGS on infant care. The full study design of the first trial, from which we report results here, has been previously published (Holm et al., 2018). In the initial trial, two cohorts of parents and newborns were recruited to participate: parents with newborns admitted to the intensive care units (ICUs) at Brigham and Women’s Hospital (BWH), Massachusetts General Hospital, and Boston’s Children’s Hospital; and parents with apparently healthy newborns admitted to the BWH Well Baby Nursery. Each family was randomly assigned to receive either the standard NBS and a detailed family history report only (control group), or the same plus their infant’s exome sequencing report (nGS group). The exome sequencing report included monogenic disease risk results, i.e., pathogenic or likely pathogenic variants in approximately 1000 genes associated with actionable or non-actionable childhood-onset conditions. Carrier status for recessive conditions was also returned. Monogenic disease risk results on highly actionable adult-onset conditions with available prevention strategies or treatment options that could impact outcome (as per the ACMG SF v2.0 list), as well as pharmacogenomic variants relevant during childhood, were also returned (Kalia et al., 2017). Adult-onset conditions were not included in the original study protocol but were later added in response to ethical concerns that arose around withholding actionable findings that may benefit the child by benefitting the parents or other family members (Holm et al., 2019). For participants enrolled after the protocol change, accepting results on actionable adult-onset conditions was a condition for enrollment. Participants who were enrolled prior to the change were contacted and given the option to consent to receive results related to adult-onset conditions (Holm et al., 2019). Reports were disclosed to families by a genetic counselor associated with the study before the reports were integrated into the electronic medical record and sent to pediatricians. Throughout the study, parents were surveyed on their experiences and their perspectives on the value of nGS. Surveys were administered at baseline, immediately post-disclosure, 3 months post-disclosure, and 10 months post-disclosure.

While the initial protocol involved limited recruitment of parents whose infants were in the ICUs, here we present findings only from surveys administered to parents enrolled from the healthy baby cohort, as this group is likely to be most representative of the general population of parents for whom population-based screening *via* nGS, to augment NBS, would be relevant. At baseline and 3 months post-disclosure, we assessed parental attitudes regarding whether every baby should receive NBS and GS at birth, whether informed consent should be required for these tests, and whether the state should require all newborns to receive GS at birth. We focus here on post-disclosure responses, as baseline survey results have been reported elsewhere, and this allows parents’ responses to be informed by their participation in the study (Pereira et al., 2019). Further, we examine attitudes assessed at 3 months post-disclosure regarding the types of results parents would want to receive from GS.

Available data from both parents of a newborn from the first trial were included in the analytic data set, and missing values were not imputed. Baylor College of Medicine’s Institutional Review Board (IRB), The Partners (now Mass General Brigham) Human Research Committee, and Boston Children’s IRB approved all aspects of the BabySeq Project. This trial is registered at ClinicalTrials.gov (NCT02422511). The data supporting the assertions of this article will be made available by the authors upon request.

### Measures

Parental attitudes regarding whether all newborns should receive, and whether informed consent should be required for, NBS and nGS, as well as whether nGS should be mandated, were assessed using five items in both the baseline and the 3-month post-disclosure surveys. This section of each survey began with a description of NBS and nGS. Novel survey items were designed to assess whether parents’ agreed or disagreed with the following statements: *1*) every newborn should receive standard NBS, *2*) parental informed consent should be required for standard NBS, *3*) every newborn should receive genomic sequencing, *4*) parental informed consent should be required for genomic sequencing of a newborn, and *5*) the state should require that all newborns receive genomic sequencing. Responses were collected on a 5-point Likert-type scale (“agreement scale”) from strongly disagree (=1) to strongly agree (=5).

Additionally, all parents were asked at 3 months post-disclosure how interested they would be in receiving the following types of information about their baby outside of the BabySeq Project, for example with their doctor or *via* a third-party service as a non-research participant. Options included: *1*) diseases that develop during *childhood* that can be prevented, treated or cured (i.e., actionable); *2*) diseases that develop during *childhood* that can NOT be prevented, treated, or cured, (i.e., non-actionable); *3*) diseases that develop during *adulthood* that can be prevented, treated, or cured; 4) diseases that develop during *adulthood* that can NOT be prevented, treated, or cured; *5*) carrier status, and *6*) variants of uncertain significance (VUSs). Carrier status was defined for parents as “information about genetic changes that my baby may have that would not cause disease in my baby but that he/she could potentially pass on to his or her own future children, or that could affect my other children.” A VUS was defined for parents as “information that the researchers or doctors have not seen before or do not fully understand.” For each type of information, parents were asked to indicate their interest on a 5-point Likert-type scale (“interest scale”) from not at all interested (=1) to very interested (=5).

If a parent agreed or strongly agreed that every newborn should receive GS at 3 months post-disclosure, they were asked to indicate whether results in each of the categories described above (actionable and non-actionable childhood onset conditions, actionable and non-actionable adult-onset conditions, carrier status, and VUS) should be returned to parents, with multiple selections possible. This question was designed to assess parents’ views on which results they felt were appropriate to include in screening reports to all newborn parents after mandated nGS screening, as this may differ from the types of results they would want for their own child (asked of all parents, as described in the previous paragraph).

We collected information about parents’ demographic characteristics at baseline. Parents’ political orientation was measured at 3 months post-disclosure using the 11-point political orientation scale from 0 to 10 with labels of Liberal (=0), Moderate (=5), and Conservative (=10) (Kroh 2007).

### Data Analysis

Descriptive statistics were calculated for parents’ demographic characteristics (at baseline and 3 months post-disclosure) and survey responses at 3 months post-disclosure. Responses to the 5-point agreement and interest scales were analyzed using Wilcoxon rank sum tests to compare parents’ level of agreement with statements or interest in receiving various types of information from genomic sequencing between parents of families who were randomized to the control and nGS groups. For each attitude question asked at 3 months post-disclosure, we used paired sample t-tests to compare parents’ responses regarding NBS to those regarding nGS. Additionally, attitudes assessed at 3-month post-disclosure were analyzed on the 5-point agreement scale by randomization arm and political orientation using Fisher’s exact tests. To facilitate analysis, we combined responses on the political orientation scale to create three categories: liberal (0–3 on original scale), moderate (4–6), or conservative (7–10). We used repeated measures analysis of variance (ANOVA) to assess the effect of randomization arm on parents’ attitudes regarding whether every newborn should receive each test at birth, whether informed consent should be required for each test, and whether the state should require that all newborns receive genomic sequencing at birth from baseline to 3 months post-disclosure. For ANOVA, Survey responses on the 5-point agreement scale were combined for “disagree” and “strongly disagree” (=1) and “agree” and “strongly agree” (=3) and analyzed on a 3-point scale with neither agree nor disagree as the midpoint (=2).

## Results

A total of 406 parents of 257 healthy newborns were enrolled in the healthy baby cohort and responded to demographic questions in the baseline survey (Pereira et al., 2019). Among these parents, 248 parents of 174 healthy newborns also submitted a survey at 3 months post-disclosure. Demographic characteristics did not differ between parents who responded at baseline and who responded at 3 months post-disclosure, except for educational attainment; a higher proportion of parents who responded at both time points had a bachelor’s degree or higher (93%), compared to those who only responded at baseline (86%; *p* = 0.028). Table 1 presents self-reported characteristics of parents who responded at 3 months post-disclosure. Thirty parents who responded to the 3 months post-disclosure survey did not respond to the baseline survey, and therefore their demographic characteristics are not available.

**TABLE 1:** Demographic characteristics of parents who completed baseline and 3 months post-disclosure surveys.

	Control (*n* = 106)	nGS (*n* = 142)	Total (*n* = 248)	*p*-value
Gender				0.318
Female	62 (58.5%)	74 (52.1%)	136 (54.8%)	—
Male	44 (41.5%)	68 (47.9%)	112 (45.2%)	—
Race				0.299
Asian	8 (8.1%)	18 (14.1%)	26 (11.5%)	—
Black or African American	4 (4.0%)	2 (1.6%)	6 (2.6%)	—
More than one race	4 (4.0%)	2 (1.6%)	6 (2.6%)	—
Other	3 (3.0%)	2 (1.6%)	5 (2.2%)	—
White	80 (80.8%)	104 (81.2%)	184 (81.1%)	—
Ethnicity				0.098
Non-Hispanic	82 (90.1%)	115 (95.8%)	197 (93.4%)	—
Hispanic or Latino	9 (9.9%)	5 (4.2%)	14 (6.6%)	—
Education level				0.892
Less than Bachelor’s	7 (6.6%)	10 (7.0%)	17 (6.9%)	—
Bachelor’s or higher	99 (93.4%)	132 (93.0%)	231 (93.1%)	—
Household income				0.334
$0–$99,999	18 (17.1%)	19 (13.7%)	37 (15.2%)	—
≥ $100,000–199,999	47 (44.8%)	54 (38.8%)	101 (41.4%)	—
≥ $200,000	40 (38.1%)	66 (47.5%)	106 (43.4%)	—
Patient is parents’ first child				0.133
No	48 (50.5%)	53 (40.5%)	101 (44.7%)	—
Yes	47 (49.5%)	78 (59.5%)	125 (55.3%)	—
Monogenic disease risk finding				
No monogenic disease risk	N/A	127 (89.4%)	127 (89.4%)	N/A
Monogenic disease risk finding	N/A	15 (10.6%)	15 (10.6%)	N/A
nGS, newborn genomic sequencing. NA, not applicable				

### Parental Attitudes Regarding Standard NBS and nGS

Table 2 presents parents’ attitudes regarding standard newborn screening and newborn genomic sequencing by study arm at 3 months post-disclosure. A majority of parents in both the control arm (96/122, 78.7%) and in the nGS arm (115/162, 71.0%) strongly agreed that every newborn should receive NBS. There was not a statistically significant interaction between the effect of study arm and time on agreement that every newborn should receive NBS (F(1, 250) = 0.20, *p* = 0.655). Average agreement among parents that every newborn should receive standard NBS (mean 4.67) was higher than that every newborn should receive GS (mean 3.60; *p* < 0.001). At 3 months post-disclosure, 18.5% (23/124) of parents in the control arm and 16.7% (27/162) of parents in the nGS arm strongly agreed that every newborn should receive nGS. There was no statistically significant interaction between study arm and time on agreement that every newborn should receive nGS (F(1, 252) = 0.66, *p* = 0.416). Parents’ average agreement that informed consent should be required to perform NBS (mean 3.44) was lower than that for nGS (mean 4.27, *p* < 0.001). At 3 months post-disclosure, 29.0% (36/124) of parents in the control arm and 26.5% (43/162) of parents in the nGS arm strongly agreed that parental informed consent should be required for NBS, while 49.2% (61/124) of parents in the control arm and 44.7% (72/161) of parents in the nGS arm strongly agreed that parental informed consent should be required for nGS. There was not a statistically significant interaction between study arm and time on agreement that informed consent should be required for either NBS (F(1, 251) = 0.52, *p* = 0.470) or for nGS (F(1, 250) = 0.07, *p* = 0.794).

**TABLE 2:** Parents’ attitudes regarding standard newborn screening and newborn genomic sequencing by study arm.

	Control	nGS	Total	*p*-value
**Every newborn should receive standard newborn screening**	*n* = 122	*n* = 162	*n* = 284	0.652
Strongly disagree	0 (0.0%)	1 (0.6%)	1 (0.4%)	—
Disagree	1 (0.8%)	2 (1.2%)	3 (1.1%)	—
Neither agree nor disagree	4 (3.3%)	7 (4.3%)	11 (3.9%)	—
Agree	21 (17.2%)	37 (22.8%)	58 (20.4%)	—
Strongly agree	96 (78.7%)	115 (71.0%)	211 (74.3%)	—
**Every newborn should receive genomic sequencing**	*n* = 124	*n* = 162	*n* = 286	0.435
Strongly disagree	3 (2.4%)	7 (4.3%)	10 (3.5%)	—
Disagree	10 (8.1%)	14 (8.6%)	24 (8.4%)	—
Neither agree nor disagree	43 (34.7%)	42 (25.9%)	85 (29.7%)	—
Agree	45 (36.3%)	72 (44.4%)	117 (40.9%)	—
Strongly agree	23 (18.5%)	27 (16.7%)	50 (17.5%)	—
**The state should require that all newborns receive genomic sequencing at birth**	*n* = 123	*n* = 159	*n* = 282	0.654
Strongly disagree	11 (8.9%)	13 (8.2%)	24 (8.5%)	—
Disagree	28 (22.8%)	41 (25.8%)	69 (24.5%)	—
Neither agree nor disagree	47 (38.2%)	52 (32.7%)	99 (35.1%)	—
Agree	23 (18.7%)	39 (24.5%)	62 (22.0%)	—
Strongly agree	14 (11.4%)	14 (8.8%)	28 (9.9%)	—
**Parental informed consent should be required for standard newborn screening**	*n* = 124	*n* = 162	*n* = 286	0.436
Strongly disagree	9 (7.3%)	19 (11.7%)	28 (9.8%)	—
Disagree	28 (22.6%)	26 (16.0%)	54 (18.9%)	—
Neither agree nor disagree	18 (14.5%)	30 (18.5%)	48 (16.8%)	—
Agree	33 (26.6%)	44 (27.2%)	77 (26.9%)	—
Strongly agree	36 (29.0%)	43 (26.5%)	79 (27.6%)	—
**Parental informed consent should be required for genomic sequencing**	*n* = 124	*n* = 161	*n* = 285	0.884
Strongly disagree	2 (1.6%)	4 (2.5%)	6 (2.1%)	—
Disagree	3 (2.4%)	6 (3.7%)	9 (3.2%)	—
Neither agree nor disagree	7 (5.6%)	12 (7.5%)	19 (6.7%)	—
Agree	51 (41.1%)	67 (41.6%)	118 (41.4%)	—
Strongly agree	61 (49.2%)	72 (44.7%)	133 (46.7%)	—

Parents’ opinions were divided as to whether states should require nGS in a manner similar to state mandated NBS. Overall, while 9.9% of parents strongly agreed that the state should require nGS, 8.5% strongly disagreed, and 35.1% of parents neither agreed nor disagreed. Only 11.4% (14/123) of parents in the control arm and 8.8% (14/159) of parents in the nGS arm agreed that the state should require that all newborns receive genomic sequencing at birth. There was not a statistically significant interaction between study arm and time on agreement that the state should require that all newborns receive genomic sequencing at birth (F(1, 248) = 1.74, *p* = 0.187).

Parents’ attitudes regarding NBS and nGS were not associated with self-reported political orientation (Table 3). Strong agreement that every newborn should receive standard NBS was high among self-identified liberals (77.6%), moderates (78.5%), and conservatives (74.3%; *p* = 0.187). While 26.4% of liberals, 34.2% of moderates, and 20.0% of conservatives strongly agreed that informed consent should be required for NBS (*p* = 0.359), 48.5, 50.6, and 26.7%, respectively, strongly agreed that informed consent should be required for nGS (*p* = 0.247).

**TABLE 3:** Parents’ attitudes regarding newborn screening and genomic sequencing by political orientation.

	Liberal	Moderate	Conservative	*p*-value
**Every newborn should receive standard newborn screening**	*n* = 161	*n* = 79	*n* = 30	0.187
Strongly disagree	1 (0.6%)	0 (0.0%)	0 (0.0%)	—
Disagree	2 (1.2%)	1 (1.3%)	0 (0.0%)	—
Neither agree nor disagree	4 (2.5%)	3 (3.8%)	3 (10.0%)	—
Agree	29 (18.0%)	13 (16.5%)	10 (33.3%)	—
Strongly agree	125 (77.6%)	62 (78.5%)	17 (56.7%)	—
**Every newborn should receive genomic sequencing**	*n* = 163	*n* = 79	*n* = 30	0.448
Strongly disagree	5 (3.1%)	4 (5.1%)	0 (0.0%)	—
Disagree	18 (11.0%)	6 (7.6%)	0 (0.0%)	—
Neither agree nor disagree	49 (30.1%)	22 (27.8%)	10 (33.3%)	—
Agree	67 (41.1%)	30 (38.0%)	13 (43.3%)	—
Strongly agree	24 (14.7%)	17 (21.5%)	7 (23.3%)	—
**The state should require that all newborns receive genomic sequencing at birth**	*n* = 163	*n* = 79	*n* = 30	0.354
Strongly disagree	10 (6.1%)	9 (11.4%)	3 (10.0%)	—
Disagree	40 (24.5%)	20 (25.3%)	7 (23.3%)	—
Neither agree nor disagree	66 (40.5%)	20 (25.3%)	9 (30.0%)	—
Agree	34 (20.9%)	19 (24.1%)	7 (23.3%)	—
Strongly agree	13 (8.0%)	11 (13.9%)	4 (13.3%)	—
**Parental informed consent should be required for standard newborn screening**	*n* = 163	*n* = 79	*n* = 30	0.359
Strongly disagree	17 (10.4%)	7 (8.9%)	2 (6.7%)	—
Disagree	34 (20.9%)	14 (17.7%)	5 (16.7%)	—
Neither agree nor disagree	30 (18.4%)	7 (8.9%)	8 (26.7%)	—
Agree	39 (23.9%)	24 (30.4%)	9 (30.0%)	—
Strongly agree	43 (26.4%)	27 (34.2%)	6 (20.0%)	—
**Parental informed consent should be required for genomic sequencing of a newborn**	*n* = 163	*n* = 79	*n* = 30	0.247
Strongly disagree	4 (2.5%)	1 (1.3%)	1 (3.3%)	—
Disagree	5 (3.1%)	3 (3.8%)	1 (3.3%)	—
Neither agree nor disagree	13 (8.0%)	3 (3.8%)	1 (3.3%)	—
Agree	62 (38.0%)	32 (40.5%)	19 (63.3%)	—
Strongly agree	79 (48.5%)	40 (50.6%)	8 (26.7%)	—

### Parent Preferences on Results

At 3 months post-disclosure, parents indicated their interest in receiving several possible types of GS results for their baby if their baby were to receive GS outside of the BabySeq Project (Table 4). A majority of parents reported being very interested in receiving information on their baby’s risk of developing a disease in *childhood* that can be prevented, treated, or cured (86.8%); risk of developing a disease during *childhood* that can NOT be prevented, treated, or cured (50.7%), baby’s risk of developing a disease during *adulthood* that can be prevented, treated, or cured (84.6%); and carrier status (70.8%). Only 42.0% of parents reported being very interested in receiving VUS results, and only 47.7% reported being very interested in learning their baby’s risk of developing a disease during *adulthood* that can NOT be prevented, treated, or cured. There were no differences in interest levels for receiving any result type between the control and nGS group (all *p* > 0.144).

**TABLE 4:** Parents’ attitudes regarding desired results from newborn genomic sequencing by study arm.

	Control	nGS	Total	*p*-value
**My baby’s risk of developing a disease during childhood that can be prevented, treated, or cured**	*n* = 124	*n* = 163	*n* = 287	0.809
Not at all interested	1 (0.8%)	0 (0.0%)	1 (0.3%)	—
Not very interested	1 (0.8%)	1 (0.6%)	2 (0.7%)	—
Neutral	4 (3.2%)	5 (3.1%)	9 (3.1%)	—
Somewhat interested	11 (8.9%)	15 (9.2%)	26 (9.1%)	—
Very interested	107 (86.3%)	142 (87.1%)	249 (86.8%)	—
**My baby’s risk of developing a disease during childhood that can NOT be prevented, treated, or cured**	*n* = 125	*n* = 163	*n* = 288	0.201
Not at all interested	7 (5.6%)	5 (3.1%)	12 (4.2%)	—
Not very interested	9 (7.2%)	9 (5.5%)	18 (6.2%)	—
Neutral	15 (12.0%)	17 (10.4%)	32 (11.1%)	—
Somewhat interested	35 (28.0%)	45 (27.6%)	80 (27.8%)	—
Very interested	59 (47.2%)	87 (53.4%)	146 (50.7%)	—
**My baby’s risk of developing a disease during adulthood that can be prevented, treated, or cured**	*n* = 124	*n* = 161	*n* = 285	0.976
Not at all interested	1 (0.8%)	0 (0.0%)	1 (0.4%)	—
Not very interested	1 (0.8%)	2 (1.2%)	3 (1.1%)	—
Neutral	3 (2.4%)	4 (2.5%)	7 (2.5%)	—
Somewhat interested	14 (11.3%)	19 (11.8%)	33 (11.6%)	—
Very interested	105 (84.7%)	136 (84.5%)	241 (84.6%)	—
**My baby’s risk of developing a disease during adulthood that can NOT be prevented, treated, or cured**	*n* = 124	*n* = 161	*n* = 285	0.144
Not at all interested	7 (5.6%)	10 (6.2%)	17 (6.0%)	—
Not very interested	18 (14.5%)	8 (5.0%)	26 (9.1%)	—
Neutral	11 (8.9%)	19 (11.8%)	30 (10.5%)	—
Somewhat interested	34 (27.4%)	42 (26.1%)	76 (26.7%)	—
Very interested	54 (43.5%)	82 (50.9%)	136 (47.7%)	—
**Carrier status**	*n* = 125	*n* = 163	*n* = 288	0.556
Not at all interested	2 (1.6%)	2 (1.2%)	4 (1.4%)	—
Not very interested	2 (1.6%)	2 (1.2%)	4 (1.4%)	—
Neutral	10 (8.0%)	7 (4.3%)	17 (5.9%)	—
Somewhat interested	24 (19.2%)	35 (21.5%)	59 (20.5%)	—
Very interested	87 (69.6%)	117 (71.8%)	204 (70.8%)	—
**Variants of uncertain significance**	*n* = 125	*n* = 161	*n* = 286	0.967
Not at all interested	4 (3.2%)	9 (5.6%)	13 (4.5%)	—
Not very interested	13 (10.4%)	10 (6.2%)	23 (8.0%)	—
Neutral	25 (20.0%)	33 (20.5%)	58 (20.3%)	—
Somewhat interested	30 (24.0%)	42 (26.1%)	72 (25.2%)	—
Very interested	53 (42.4%)	67 (41.6%)	120 (42.0%)	—

**FIGURE 1:**
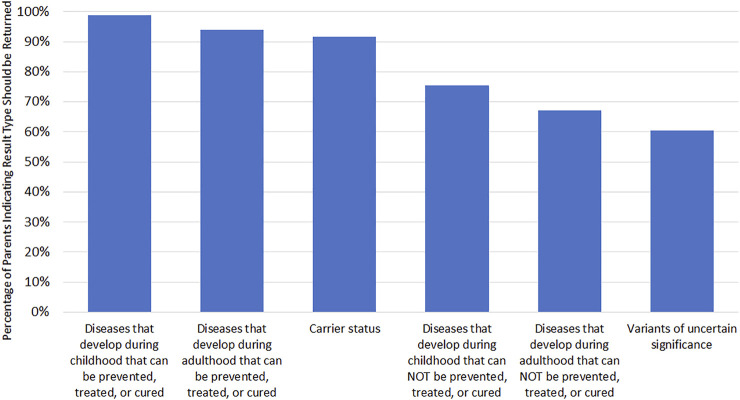
Attitudes toward results types to be returned to parents if every newborn received GS. Only asked if parent agreed or strongly agreed that every newborn should receive GS (*n* = 167). Respondents could select multiple options.

Among parents who strongly agreed or agreed that every newborn should receive nGS at 3 months post-disclosure (*n* = 167), the most frequently selected categories of findings that should be returned to parents were actionable findings in childhood (98.8%) and adulthood (94.0%; Figure 1).

## Discussion

In this analysis of survey responses from parents of healthy newborns in the BabySeq Project, parents were more supportive of every newborn receiving NBS than receiving nGS. We found no significant difference in nGS support between parents in the control arm and parents who had experienced receiving nGS results for their newborn, and results suggest that the experience of receiving nGS results did not affect parents’ attitudes over time. While a majority of parents supported the notion that every newborn should receive GS, only a minority thought that the state should require nGS. Additionally, a larger proportion of parents agreed that parental informed consent should be required for nGS than for standard NBS. Previous studies examining parent attitudes toward standard NBS and nGS have also reported parent concern about not requiring informed consent for nGS, increased parent support for standard NBS compared to nGS, and disagreement between parents about which results should be reported (Bombard et al., 2014; Joseph et al., 2016; Lewis et al., 2018; Moultrie et al., 2020). Though most parents indicated they would be interested in receiving all available result types if their baby received GS outside the BabySeq Project, enthusiasm varied among result types.

NGS in newborns may provide health benefits and information complementary to standard NBS. A previously published study from the BabySeq Project showed that families experienced no sustained negative psychosocial effects from participating in the GS process or receiving results, a concern frequently raised in the discussion surrounding the addition of nGS (Pereira et al., 2021; Wojcik et al., 2021). However, even highly successful programs like standard NBS can come under scrutiny if policies are not acceptable to parents (National Institute of Child Health and Human Development 2017). It is critical to consider parent buy-in before implementing policies that impact NBS programs; not doing so may risk negatively affecting parent trust, participation, and thus the overall success of the program.

Even among our group of parents who had enough interest in nGS to volunteer to participate in the BabySeq Project, a majority of parents thought informed consent should be required for nGS and many were still hesitant about adding nGS to current state mandated NBS programs. Considering that parents who participated in the BabySeq Project may likely be more supportive of nGS than the average parent, our study results suggest that implementing nGS without addressing parental concerns could create parent backlash. Another study comparing parent views on nGS vs standard NBS in Canada came to a similar conclusion after finding parents were significantly less willing to participate in a NBS program that included whole genome or exome sequencing (Bombard et al., 2014). Notably, a majority of our parents thought that informed consent should also be required for standard NBS, which is not legally required in most states. It may be possible that, although parents may ideally want an informed consent process, they tolerate a lack of informed consent because such a strong majority feel that every newborn should receive standard NBS. However, nGS does not share this same level of support in our study sample.

One approach to accommodate parents’ preferences while preserving participation in current newborn screening programs would be for nGS to be an optional addition to state mandated NBS that requires explicit informed consent. This optional add-on consent model gives the opportunity for counseling on GS screening to ensure parents understand the capacity, utility, and limitations of GS. This approach was successfully implemented for expanded NBS using tandem mass spectrometry. For example, when Massachusetts added mass spectrometry to their NBS program as an optional add-on program in 1999, 98% of parents chose to participate, prompting other states to expand their newborn screening programs (Marsden 2003). More recent studies examining newborn genetic screening for SMA and Duchenne Muscular Dystrophy have also used this model and experienced high parent participation rates (Kraszewski et al., 2017; Parad et al., 2021). While an optional add-on model could help demonstrate the health benefits of GS screening without compromising existing mandated public health programs, it induces the burden of additional informed consent and documentation on hospital staff. One California study examining the introduction of mass spectrometry to NBS that required informed consent demonstrated significant burden of documentation, resulting in many families not being offered the additional screening (Feuchtbaum et al., 2007). If hospital systems are not prepared to incur the burden of additional screening, increased documentation, follow-up and parent counseling that would be required to incorporate nGS into NBS, the addition of these programs may fail to produce the desired result and overall compromise parent satisfaction and trust. There are also concerns that requiring informed consent for any portion of the NBS may reduce overall participation rates (Davis et al., 2006; Feuchtbaum et al., 2007).

Interestingly, while other studies have found some association between political orientation and interest in genomic sequencing (Dodson et al., 2015), political orientation was not significantly associated with opinions of whether states should require GS in our study. This suggests that it may be possible to garner bipartisan support for policies regarding nGS. Finally, there is the issue of what results should be returned to parents. In our study, there was variation among parents on which nGS results they would want to receive. Differences in parent preferences may best be supported by an informed consent model that incorporates parental choice about the return of results, although this would likely be highly burdensome to NBS programs. Parent preferences may also not align with what results professional guidelines deem ethically justified to report for minors. NBS mandates are justified on the ethical basis that screening in the newborn period provides the opportunity to initiate early intervention after birth to prevent harm, and they are justified on the legal basis that significant public health benefits provide a compelling government interest. To maintain this justification, genetic testing results should only be disclosed if there is clear clinical value (Ross et al., 2013; Botkin et al., 2015). However, not all GS results have the promise of early or even certain direct benefit to the child being tested (Timmermans and Buchbinder 2010; Berg and Powell 2015; Johnston et al., 2018; Lewis 2019). The contrast between which results are considered ethical to return and which results parents want may pose challenges if whole genome or exome sequencing is used for nGS, as parents may be able to invoke a legal right to the entirety of their child’s genomic data.

Our results should be considered within the limitations of our study. Study participants were parents willing to participate in a genomics study from three hospitals in the Boston, Massachusetts area. As such, opinions may differ significantly between study participants and the general population. It is also important to note that our study demographics are not representative of the general US population, with a high proportion of non-Hispanic white individuals, high household income, and high educational attainment. Representative surveys are warranted to provide more generalizable information suited to inform federal and state policy discussions. The second iteration of the BabySeq Project, BabySeq2, currently underway, will prioritize the inclusion of a more racially, ethnically, and geographically diverse cohort of families (https://www.genomes2people.org/research/babyseq/) and will provide additional data on parents’ attitudes. Finally, our surveys were not designed to capture nuanced views; it is possible that parents may have expressed more tempered attitudes toward screening and results types in interviews or focus groups.

Currently, the NBS program has parents’ trust and near universal participation. Any policies created to expand the NBS program to include nGS should strive to protect this trust and preserve parent support by considering parent values. We propose that should nGS be added to current NBS programs, parent values could be respected if it were initially added as an optional supplemental screen that requires an informed consent process with preservation of the default mandatory NBS using traditional methods.

## BabySeq Team Members

Pankaj B Agrawal, Alan H. Beggs, Wendi N. Betting, Ozge Ceyhan-Birsoy, Kurt D. Christensen, Dmitry Dukhovny, Shawn Fayer, Leslie A. Frankel, Casie A. Genetti, Chet Graham, Robert C. Green, Amanda M. Gutierrez, Maegan Harden, Ingrid A Holm, Joel B. Krier, Matthew S. Lebo, Kaitlyn B. Lee, Harvey L. Levy, Xingquan Lu, Kalotina Machini, Amy L. McGuire, Jaclyn B. Murry, Medha Naik, Tiffany T. Nguyen Dolphyn, Richard B. Parad, Hayley A. Peoples, Stacey Pereira, Devan Petersen, Uma Ramamurthy, Vivek Ramanathan, Heidi L. Rehm, Amy Roberts, Jill Oliver Robinson, Sergei Roumiantsev, Talia S. Schwartz, Hadley Stevens Smith, Tina K Truong, Grace E. VanNoy, Susan E. Waisbren, Timothy W. Yu, Carrie L Blout Zawatsky, Bethany Zettler.

## Data Availability

The raw data supporting the conclusions of this article will be made available by the authors, without undue reservation.
